# Effect of lentivirus-mediated survivin transfection on the morphology and apoptosis of nucleus pulposus cells derived from degenerative human disc *in vitro*

**DOI:** 10.3892/ijmm.2015.2225

**Published:** 2015-05-26

**Authors:** XUEXIAO MA, YAZHOU LIN, KUN YANG, BIN YUE, HONGFEI XIANG, BOHUA CHEN

**Affiliations:** 1 Department of Orthopedic Surgery, The Affiliated Hospital of Qingdao University Medical College, Qingdao University, Qingdao, Shandong 266003, P.R. China; 2 Medical Research Center, The Affiliated Hospital of Qingdao University Medical College, Qingdao University, Qingdao, Shandong 266003, P.R. China

**Keywords:** nucleus pulposus derived from human degenerative disc, lentivirus, transfection, survivin, morphology, apoptosis

## Abstract

Lower back pain is a common concern, and 40% of all cases involve the degeneration of the intervertebral disc (IVD). However, the excessive apoptosis of disc cells plays an important role in IVD degeneration, particularly in the nucleus pulposus (NP). Thus, anti-apoptotic gene therapy to attenuate or reverse the degenerative process within the NP is being developed. Survivin is a unique inhibitor of apoptosis (IAP) and has been extensively investigated in cancer cells. However, little is known of the effects of survivin transfection on NP cells derived from degenerative human disc. In this study, we aimed to investigate the effects of lentivirus (LV)-mediated survivin transfection on the morphology and apoptosis of NP cells derived from degenerative human disc *in vitro*. NP cells were transfected with LV-mediated survivin. Subsequently, cell morphology was observed and the survivin mRNA expression levels were measured by RT-qPCR. Apoptosis was analyzed by flow cytometry and by measuring caspase-3 activity. The results revealed that the morphology of the NP cells derived from degenerative human disc transfected with LV-mediated survivin was significantly altered as evidenced by cytomorphosis, the reduction of the cytoplasm and cell shrinkage. Following transfection, survivin gene expression significantly increased in the transfected cells and subsequent generation cells; however, no significant differences in the cell apoptotic rate and caspase-3 activity were observed. We found that transfection of the survivin gene into NP cells led to the stable expression of survivin and induced marked changes in cell morphology. Furthermore, no significant anti-apoptotic effects were observed following LV-mediated survivin transfection. Overall, our findings demonstrate that LV carrying surviving may be used to successfully enforce the expression of survivin in NP cells. However, cell morphology was evidently altered, whereas the apoptotic rate did not decrease. Comprehensive studies on the feasibility of using survivin in gene therapy in an aim to attenuate disc degeneration are warranted. Further research on the mechanisms responsible for the changes in cell morphology and cell function are also required.

## Introduction

Lower back pain is an endemic problem that causes substantial disability (1–3). It is estimated that 60–80% of individuals are affected by lower back pain at some point in their lives (4–7). The causes of lower back pain are multifactorial, although 40% of all cases involve the degeneration of the intervertebral disc (IVD) (8). However, conservative and surgical treatments only target the alleviation of the symptoms rather than the repair or deceleration of the underlying degenerative process. Therapies, such as gene therapy, to attenuate or reverse the degenerative process within the IVD are being developed (9).

The IVD consists of two regions: the inner nucleus pulposus (NP) and the outer anulus fibrosus (AF). The normal NP is mainly composed of aggrecan and collagen type II, whereas the normal AF contains significant amounts of collagen type I (10–12). Previous studies have demonstrated that the number of NP cells decreases and the composition of the extracellular matrix associated with these cells is altered in degenerative discs (13–15). *In vitro* and *in vivo* studies have suggested that the cellular loss attributed to the excessive apoptosis of disc cells plays an important role in IVD degeneration (16,17).

Among the apoptotic signaling pathways, two main caspase-dependent pathways have been observed: the intrinsic and extrinsic pathways, which are mediated by the mitochondria and death receptor, respectively (18). Several studies have demonstrated that the mechanism of apoptosis involves two pathways in NP cells derived from degenerative human disc which vary among patients. However, these two pathways ultimately induce caspase-3 to initiate apoptosis (19–22).

**Figure 1:**
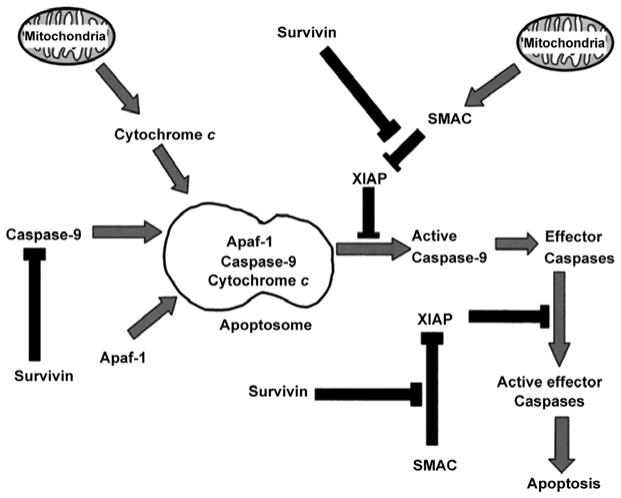
Schematic diagram of the mechanisms of survivin-mediated anti-apoptotic pathways.

Survivin is a unique inhibitor of apoptosis (IAP) that deters the activation of intrinsic and extrinsic pathways, with a focus on the former. Survivin inhibits apoptosis by binding to caspase-9 or by blocking second mitochondria-derived activator of caspases (SMAC; a pro-apoptotic protein that binds IAPs and thus prevents them from inhibiting caspases) and thus prevents the pro-apoptotic protein from blocking IAP proteins (Fig. 1) (23). The expression of survivin is strictly controlled in embryonic tissues and in the majority of tumors, but not during tissue differentiation and maturation (23,24). Thus, survivin presents an attractive target for cancer therapy (25), and has been extensively studied in cell cycle and apoptotic assays for cancer cells (26,27). Studies have demonstrated that the oncofetal gene, survivin, is re-expressed in osteoarthritis and rheumatoid arthritis (28–30). Moreover, preliminary studies have indicated that survivin is expressed in fetal disc tissue and have noted the differential expression of survivin between NP tissue derived from degenerative disc and that derived from a relatively normal disc (31,32). However, to our knowledge, limited research has been conducted on the effects of lentivirus (LV)-mediated survivin transfection on NP cells derived from degenerative human disc *in vitro*.

The present study was conducted to determine the effects of the LV-mediated survivin transfection on the morphology and apoptosis of NP cells *in vitro*. Our results provide fundamental information on the effectiveness of gene therapy aimed at the attenuation of disc degeneration *in vitro*.

## Materials and methods

### Experimental materials

NP samples from herniated IVDs of the lumbar spine were collected from 10 patients, including 6 males and 4 females, with a mean age of 51 years (46–62 years) (Table I). All patients were diagnosed with IVD degeneration (lumbar disc herniation) by magnetic resonance imaging and had undergone spinal fusion to relieve chronic lower back pain. This study was approved by the Ethics Committee of the Affiliated Hospital of Qingdao University Medical College (Shandong, China). Written informed consent was obtained from all patients prior to enrollment; all patients agreed to the publication of their clinical data.

The tissue material was harvested and collected under sterile conditions. A phosphate-buffered saline (PBS) solution at 4°C was used as a transport medium. All biopsies were delivered to the laboratory for the processing of cell culture on the day of harvest.

LV with survivin and an empty LV were purchased from Shanghai Genechem Co. Ltd. (Shanghai, China) The titers of the LV carrying survivin and the empty LV were 2×10^8^ and 1×10^9^ TU/ml, respectively.

### Cell isolation and culture

The tissues were weighed and washed twice in PBS. The NP and AF were separated based on their macroscopic morphologies by omitting the transitional zone. Cells from each patient were isolated and separately cultured. The NP tissue was cut into small sections (approximately 1 mm^2^) and then digested with 0.25% trypsinase (HyClone, Logan, UT, USA) at 37°C under gentle agitation. After 20 min, the digestion was terminated using DMEM/F12 medium (HyClone) with 15% fetal calf serum (Gibco-BRL, Carslbad, CA, USA) and the tissues were centrifuged at 1,000 rpm for 5 min. Subsequently, 0.5% collagenase type II (MP Biomedicals, LLC, Santa Ana, CA, USA) was used at 37°C for approximately 4 h, after which the tissues were centrifuged at 1,000 rpm for 5 min and washed thrice with DMEM/F12 medium containing 15% fetal calf serum.

The cells were transferred to a 12.5-cm^2^ culture flask at a density of 10^5^ cells/cm^2^. The cells were then cultured in a CO_2_ incubator (Sanyo Electric Co., Ltd., Osaka, Japan) at 37°C with humidity and then grew in DMEM/F12 containing 15% fetal calf serum and 1% penicillin/streptomycin (Invitrogen Life Technologies, Tokyo, Japan). The growth medium was changed every 3 days after cell adhesion. Cell samples from different patients were kept separate from one another. All experiments were carried out in duplicate and were conducted with human NP cells from passages 2 to 3.

### Immunohistochemistry

NP cells from passage 2 were cultured on glass slides and were then fixed for 10 min with 4% paraformaldehyde followed by permeabilization for 5 min with 0.1% Triton X-100 in PBS. For antigen retrieval, the slides were boiled for 20 min (10 mM citrate buffer, pH 6.0) and then endogenous peroxidase was Affiliated Hospital of Qingdao University Medical College blocked using hydrogen peroxide. The cells were pre-incubated for 1 h in a solution of PBS containing 10% goat serum in order to prevent non-specific antibody binding. The NP cells were incubated for 16 h at 4°C with anti-human collagen type II antibody [1:100; Cat. no. ab3092) and anti-human aggrecan antibody (1:100; Cat. no. ab3778) [both from Abcam (Hong Kong) Ltd., Hong Kong, China]. Following incubation, the cells were washed 3 times with PBS, incubated for 1 h at room temperature with rabbit anti-mouse IgG and then rinsed with PBS. This was followed by coloration with DAB and hematoxylin staining and observation under a microscope (Olympus GX51; Olympus Corp., Tokyo, Japan).

### Gene transfection

To quantify the percentage of successfully transfected NP cells at a given multiplicity of infection (MOI), an identical procedure was performed with the LV-green fluorescent protein (GFP) (Shanghai Genechem Co., Ltd.) for each MOI assessed. NP cells from passage 2 were plated as a monolayer in 96-well plates at 4×10^4^ cells/ml and were incubated for 24 h. Solutions of the viral particles equal to 30, 40, 60, 80 and 100 MOI were pre-mixed with DMEM/F12 medium and were added to the 96-well plates. After 48 h, the NP cells were examined under a fluorescence microscope (Olympus CKX41SF; Olympus Corp.), and the percentage of NP cells synthesizing GFP was determined.

NP cells from passage 2 were divided into 3 groups (the positive, negative control and blank control groups), which were transfected with LV with survivin, the empty LV or an equal amount of DMEM/F12 medium, respectively. The transfection procedure was performed with an MOI value of 50. The transfected NP cells were incubated in a CO_2_ incubator at 37°C. After 8 h, the growth medium was changed.

### Observation of cell morphology

The morphology and growth of the NP cells were observed daily under an inverted microscope (Olympus CKX41; Olympus Corp.). The cell morphological changes induced by transfection with LV carrying survivin were recorded and images were obtained using a microscope.

### RNA extraction and reverse transcription-quantitative PCR (RT-qPCR)

To measure the expression of survivin following transfection with LV in the transfected cells and subsequent generation cells, RT-qPCR was performed. mRNA was extracted from the NP cells using TRIzol reagent (Invitrogen Life Technologies, San Diego, CA, USA) according to the one-step method. A total of 1 *µ*g mRNA was reverse transcribed into cDNA using PrimeScript RT Reagent (Takara DRR037A; Takara Bio, Inc., Shiga, Japan), and the reaction product was treated with RNase-Free DNase I. The absorbance at 260 and 280 nm was measured for quantification and quality control.

qPCR was conducted using the following cycling conditions (LightCycler 480II; Roche Diagnostics GmbH, Mannheim, Germany): 95°C for 5 min followed by 33 cycles of 94°C for 45 sec, 56°C for 45 sec and 72°C for 45 sec and a final extension at 72°C for 10 min. Primers and probes were designed using Primer Express Software (Applied Biosystems, Ltd., Warrington, UK). Total gene specificity was confirmed by BLAST searches (GenBank database sequences). Primers were purchased from Sangon Biotech Co., Ltd. (Shanghai, China) (Table II). Another specific primer pair for human glyceraldehyde-3-phosphate dehydrogenase (GAPDH) was used as an internal control. In each experiment, samples were analyzed in duplicate. The normalized target gene expression was determined through the comparative Ct (ΔΔCt) method.

### Detection of apoptosis by flow cytometry and measurement of caspase-3 activity

Second-generation NP cells transfected with LV were placed in 6-well culture plates at 1×10^5^ cells/well and treated as above. The apoptotic rate of the NP cells was detected by Annexin V/propidium iodide (PI) (Nanjing KeyGen Biotech. Co., Ltd., Jiangsu, China) double staining according to the manufacturer’s instructions and as previously described (17,33). Briefly, the cells of the different groups were collected by trypsinization and centrifugation, and then washed with ice-cold PBS twice and resuspended in 500 *µ*l binding buffer. A total of 5 *µ*l of fluorescein-conjugated Annexin V and 5 *µ*l of PI was added followed by further incubation in the dark for 15 min at room temperature. The apoptotic rate was analyzed by flow cytometry (BD LSR II flow cytometer) using FACSDiva software (both from Becton-Dickinson, Franklin Lakes, NJ, USA). The cells stained positive for Annexin V and negative for PI were identified as early apoptotic cells and those positive for double staining were identified as late apoptotic cells in each sample. They were counted and represented as a percentage of the total cell population.

Apoptosis was evaluated by measuring caspase-3 activity using a Caspase-3 Colorimetric Assay kit (BioVision, Inc., Milpitas, CA, USA). Second-generation NP cells were counted and pelleted at 1.5×10^6^ cells for 48 h following transfection with LV carrying survivin or the empty LV. The cells were then resuspended in cell lysis buffer, and 50 *µ*l of 2X reaction buffer (containing 10 mM DTT) and 5 *µ*l of DEVE-pNA were added. The samples were incubated for 90 min at 37°C, and the absorbance was read at 405 nm using a Microtiter Plate Reader (Sunrise™; Tecan Group, Ltd., Männedorf, Switzerland).

### Statistical analysis

All values were presented as the means ± SEM. One-way ANOVA with Fisher’s least significant difference (LSD) post hoc test were applied to reveal the statistical significance of the differences. A value of P<0.05 was considered to indicate a statistically significant difference. Statistical analyses were performed using SPSS software for Windows, version 19 (SPSS, Inc., Chicago, IL, USA).

## Results

### Transfection efficiency of LVs

**Figure 2:**
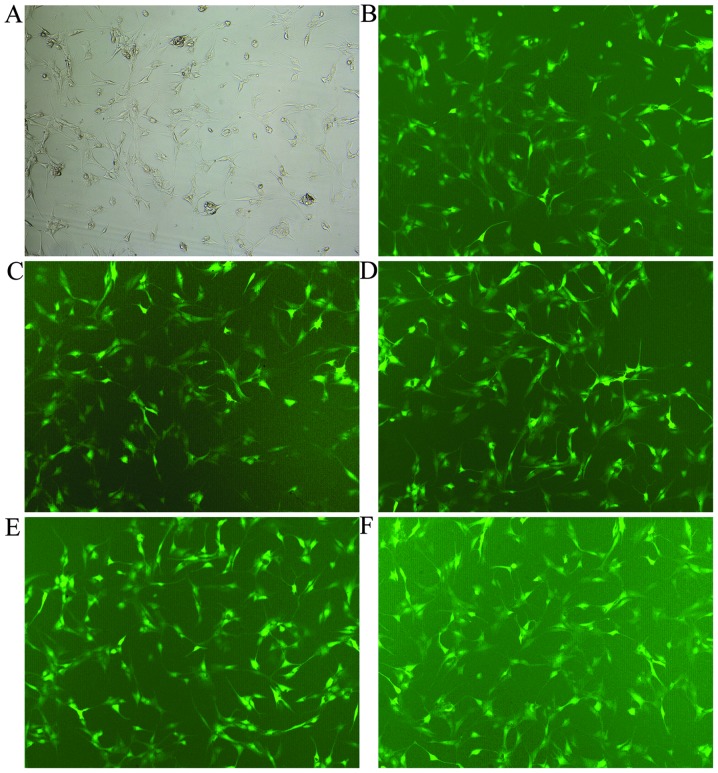
The lentivirus (LV) transfection efficiency at different multiplicities of infection (MOIs), as shown under a fluorescence microscope (magnification, ×100). (A and B) Nucleus pulposus (NP) cells were observed under an inverted microscope and a fluorescence microscope at an MOI of 60. (C–F) LV transfection efficiency at 30, 40, 80 and 100 MOI under a fluorescence microscope, respectively.

Following transfection of the NP cells with the LVs for 48 h, all the transfected cells expressed GFP successfully. For NP cells derived from degenerative human disc, the changes in the transfection rate were demonstrated by the changes in MOI (transfection with LV-GFP): 60–70% of cells with an MOI of 30 and 40, 70–85% of cells with an MOI of 60 and 80–100% of cells with an MOI of 80 and 100 (Fig. 2).

### Observation of cell morphology under a light microscope

**Figure 3:**
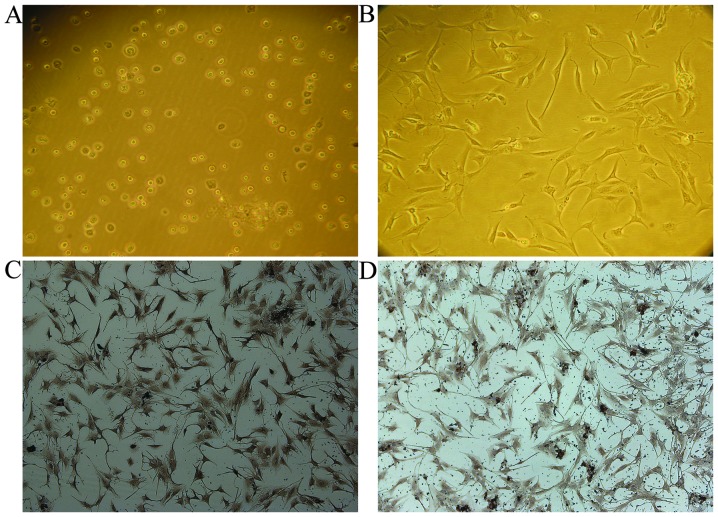
Isolation, culture and identification of primary nucleus pulposus (NP) cells. (A) Primary NP cells were obtained by enzymatic digestion (magnification, ×100). (B) Degenerated NP cells from passage 2 attached to the culture dish after passage for 3 h (magnification, ×100). The primary NP cells derived from degenerative human disc were round at the moment of isolation (A) and they had attached to the culture dish after 5–7 days of culture. The cells gradually became elongated and triangular or polygonal in shape, and the cytoplasm became plump and equally distributed. The number of attached cells exponentially increased. (C and D) Immunostaining revealed that these cells were positive for aggrecan and type II collagen, which are makers of the NP cell phenotype (magnification, ×100).

The primary NP cells derived from degenerative human disc were round at the moment of isolation (Fig. 3A) and they had attached to the culture dish after 5–7 days of culture. The cells gradually became elongated and triangular or polygonal in shape, and the cytoplasm became plump and equally distributed. The number of attached cells exponentially increased. After 15–20 days, 90% of the cells had formed colonies. The passaged NP cells derived from degenerative human disc only required 3 h to attach to the culture dish, and 90% of the cells formed colonies after 7–10 days of culture. Cell morphology was similar to that of primary cells (Fig. 3B).

To identify the NP cell phenotype, immunohistochemistry for type II collagen and aggrecan was performed (Fig. 3C and D). The results revealed that these cells expressed both type II collagen and aggrecan, which confirmed the NP cell phenotype.

**Figure 4:**
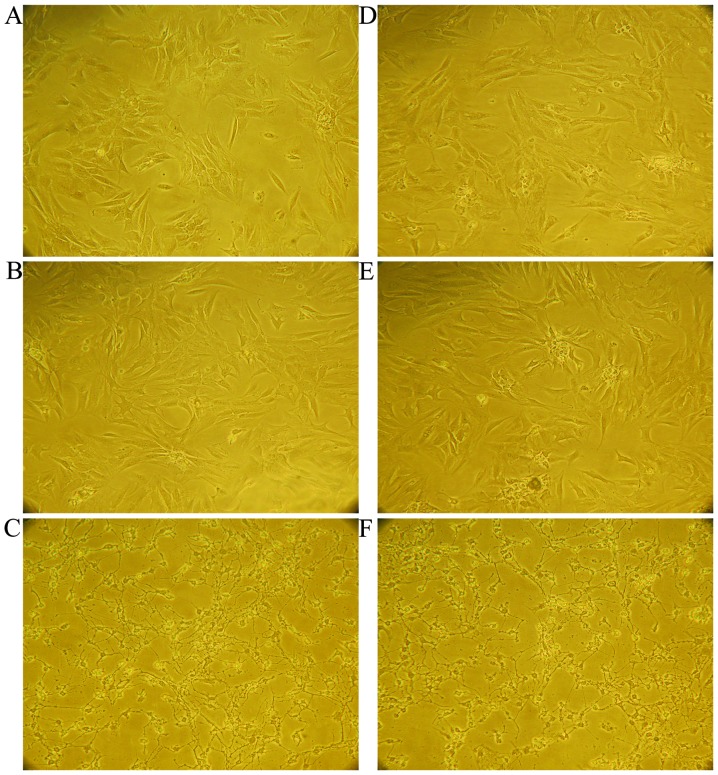
Morphology of nucleus pulposus (NP) cells derived from degenerative human disc from passage 2 and 3 (magnification, ×100). (A–C) NP cells from passage 2 following transfection for 3 days with.the blank control (equal amount of DMEM/F12 medium), negative control (empty LV) and positive control (LV with survivin), respectively. Following transfection with survivin, cytomorphosis, the reduction of the cytoplasm and cell shrinkage were evident. The cell volume changed significanlty, and pseudopodia became longer. (D–F) Using the cells described in (A–C, respectively), the cells were passaged for 1 more day. The morphology of the third-passage cells was similar to that of the second-passage cells.

In the second-passage exponential growth phase, survivin was transfected into the cells using LV. At 3 days after transfection, the morphology of the NP cells derived from degenerative human disc, which were transfected with LV carrying survivin significantly changed compared with that of the control groups. In addition, cytomorphosis, the reduction of the cytoplasm and cell shrinkage were evident. The cell volume changed significanlty, and pseudopodia became longer (Fig. 4A–C). The morphology of the third-passage cells was similar to that of the second-passage cells (Fig. 4D–F).

### Expression levels of surviving

**Figure 5:**
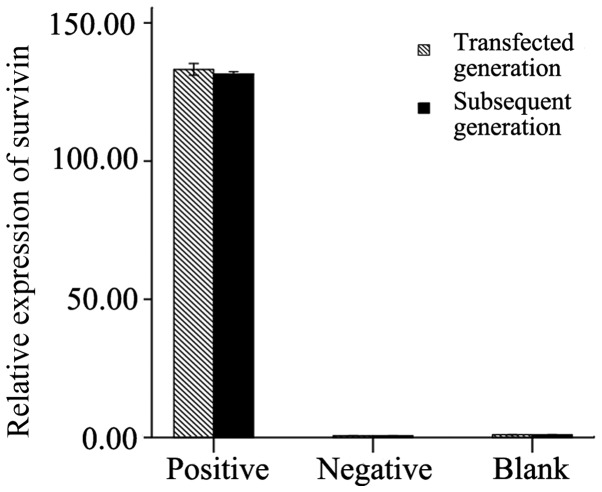
RT-qPCR of survivin mRNA expression following transfection with lentivirus (LV) carrying survivin in the transfected ccells and subsequent generation cells (passage 3). The expression of survivin following transfection was significantly higher than that in the control groups (p<0.01). No significant difference in the expression levels was observed between the transfected cells and subsequent generation cells, as well as between the negative and the blank control groups (p>0.05). Positive group, transfection with lentivirus carrying survivin; negative group, transfection with empty lentivirus; blank group, transfection with an equal amount of DMEM/F12 medium.

The survivin mRNA expression levels in the NP cells derived from degenerative human disc were measured in the transfected cells and subsequent generation cells. The results revealed that in the transfected cells and subsequent generation cells, the expression of survivin following transfection with LV carrying survivin was significantly higher than that in the control groups (p<0.01). No significant difference in the survivin expression levels was observed between the transfected cells and subsequent generation cells (transfected generation + LV-survivin vs. subsequent generation + LV-survivin, p=0.242; transfected generation + empty LV vs. subsequent generation + empty LV, p=0.996; transfected generation + equal DMEM/F12 medium vs. equal DMEM/F12 medium + equal DMEM/F12 medium, p=0.999). In additon, no significant differences were observed between the negative control and blank control groups (transfected cells, p=0.786; subsequent generation cells, p=0.790; Fig. 5).

### Measurement of apoptotic rate and caspase-3 activity

**Figure 6:**
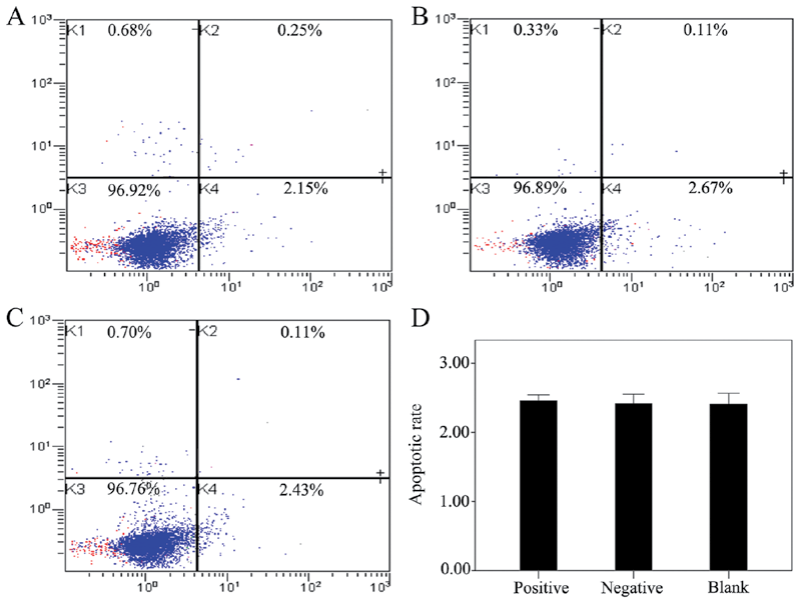
The apoptotic rate of nucleus pulposus (NP) cells following transfection with lentivirus (LV) carrying survivin was analyzed by flow cytometry. (A–C) The apoptotic rate is represented as the percentage of the total cell population. The proportion of dead cells (Annexin V^−^/PI^+^), live cells (Annexin V^−^/PI^−^), early apoptotic cells (Annexin V^+^/PI^−^) and late apoptotic/necrotic cells (Annexin V^+^/PI^+^) was measured for comparison. (A–C) NP cells from passage 2 following transfection in the positive control (LV with survivin), negative control (empty LV) or the blank control (equal amount of DMEM/F12 medium), respectively. (D) Histogram showing the results of statistical analysis shows the apoptotic rate of the NP cells following transfection (p>0.05).

**Figure 7:**
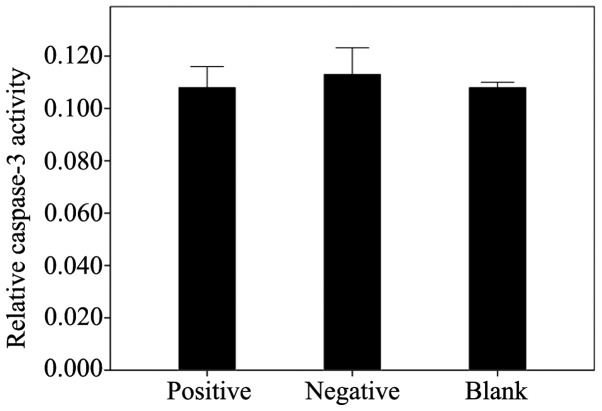
Colorimetric detection of caspase-3 activity following transfection (data are the means ± SEM). No significant differences in caspase-3 activity were observed between the groups (p=0.858). Positive group, transfection with lentivirus carrying survivin; negative group, transfection with empty lentivirus; blank group, transfection with an equal amount of DMEM/F12 medium.

The effect of LV-mediated survivin transfection on the apoptosis of the NP cells derived from degenerative human disc was examined by flow cytometry and by measuring caspase-3 activity. The results revealed that the transfection of survivin into the NP cells did not contribute to decreasing the apoptotic rate and caspase-3 activity. No significant differences in the apoptotic rate (Fig. 6) and in caspase-3 activity (Fig. 7) were observed between the positive (survivin-transfected cells and the control groups (negative and blank; apoptotic rate, p=0.952; caspase-3 activity, p=0.858).

## Discussion

Degenerative disc disease is a serious healthcare problem. Traditional methods focus on the treatment of multiple symptoms simultaneously. Gene therapy is a developing technology with great potential. The *in vitro* culture of NP cells derived from degenerative human disc serves as the foundation of gene therapy and aids in the study of cell proliferation, cell morphology, gene and protein expression, as well as in cell function. Therefore, a better understanding of the *in vitro* culture of NP cells and the identification of NP cells are important for future research.

Disc tissue consists of NP and AF cells. No significant differences have been observed in the morphology of NP and AF cells during monolayer expansion (34). However, significant differences have been found between the matrices of NP and AF cells. NP cells have a gelatinous structure that is primarily composed of aggrecan and collagen type II. The outer AF cells contain substantial amounts of collagen type I (35–37). Therefore, the current study confirmed the NP cell phenotype by using immunohistochemistry for type II collagen and aggrecan.

In our study, in primary culture the NP cells had a polygonal shape with short pseudopodia during early proliferation. However, these cells later became increasingly elongated. This change in cell morphology was even more pronounced when the cells were passaged. Compared with the primary NP cells, the passaged cells had reduced adhesiveness and mostly assumed a short spindle-shaped appearance. After the fourth passage, cells have been shown to develop slowly (34). For the negative and blank control groups, our observations were consistent with the results of a previous study (34).

However, in the positive group (survivin-transfected cells), the cell morphology was significantly altered compared with the other 2 control groups in terms of the reduction in the cytoplasm, cell shrinkage, lengthening of pseudopodia and increased intercellular space. Nevertheless, the cells did not die, but rather remained attached to the culture dish. After passage, these cells sequentially attached to the dish but did not develop. This phenomenon suggested that the cell morphology was affected by transfection.

The results obtained by RT-qPCR revealed the stable overexpression of survivin following transfection with LV carrying survivin in the transfected cells and subsequent generation cells. No significant differences were observed between the NP cells derived from degenerative human disc transfected with the empty LV and an equal amount of culture fluid (DMEM/F12). Furthermore, during our research, LV-TGFβ3, LV-TIMP1, LV-TGFβ3-TIMP1, LV-survivin-TIMP1, LV-survivin-TGFβ3, and LV-survivin-TGFβ3-TIMP1 were simultaneously transfected into NP cells derived from degenerative human disc. The results revealed that similar morphological changes occurred in the NP cells following transfection with LV-survivin-TIMP1, LV-survivin-TGFβ3, and LV-survivin-TGFβ3-TIMP1. No changes were observed in the NP cells following transfection with LV-TGFβ3, LV-TIMP1, LV-TGFβ3-TIMP1 without survivin (data not shown). These results suggest that survivin contributes to these morhological changes.

Cell morphology is at least partly determined by the cytoskeleton. It has been demonstrated that some factors may alter actin filaments through the activation or inhibition of distinct mitogen-activated protein kinase (MAPK) pathways (38). MAPK pathways have been implicated in G2/M phase regulation and apoptosis (39–42). The regulation of survivin is closely associated with MAPK pathways (23,43). Therefore, the overexpression of survivin may reversely affect MAPK pathways, similar to the inhibition of different tyrosine kinases in the actin signal transduction pathways, which may subsequently result in alterations in cytoskeleton dynamics. To confirm this phenomenon, further studies are required to examine the structure of the actin filaments in cells, in which following transfection with LV carrying survivin, the phosphorylation state of p38 MAPK, extracellular signal-regulated kinase (ERK) and JNK in response to transfection needs to be analyzed.

In previous studies, using immunohistochemical staining, survivin expression was observed in 20-, 26- and 28-week fetal-age IVDs and the differences were not statistically significant (31). Survivin was strongly expressed in NP tissue from degenerative human disc, whereas it was weakly expressed in NP cells from relatively normal disc, the difference being statistically significant (p=0.048). These previous data demonstrate that survivin plays an important role in fetal IVD growth and is extremely likely to be involved in the regulation of apoptosis and cell proliferation in the degeneration process of NP tissue (31,32). We designed the present study based on the fact that the main function of survivin (mitosis regulation and apoptosis inhibition) contributes to an increased number of NP cells, which attenuates IVD degeneration. However, the results did not meet our expectations, namely that the apoptotic rate and caspase-3 activity would decrease following LV-survivin transfection. However, no significant decrease was observed.

No significant decrease was observed in the apoptotic rate and caspase-3 activity following transfection with LV carrying survivin. This result may be attributed to the culture conditions (sufficient oxygen and glucose). As the IVD is the largest avascular organ in the body and does not directly supply blood to NP cells (44,45), material and gas exchange mainly depends on diffusion from the nearest blood supply. Owing to the progressive age-related degeneration and calcification of the cartilage end-plate (46), the number of arteries that supply the periphery of the disc decreases. This decrease impairs the diffusion function of the IVD and, therefore, nutrition to the disc and oxygen supply deteriorate. Thus, NP cells derived from degenerative human disc are under a relative ischemic condition. Thus, further studies are required to analyze the function of survivin in *in vitro* culture under ischemic conditions.

In the present study, we used LV vectors to transfect survivin into NP cells and analyzed the gene expression of survivin by RT-qPCR. Changes in cell morphology were closely observed under a microscope and recorded. Apoptosis was evaluated assessed by performing flow cytometry and by measuring caspase-3 activity. It is our intention to further study cell morphology using electron microscopy in order to confirm the changes in cell ultrastructure. and explore the effect of transfection with LV carrying survivin on the apoptosis of NP cells derived from degenerative human disc in ischemia culture for comparison with normal culture conditions.

In conclusion, we demonstrated that LV carrying survivin may be used to effectively deliver the survivin gene into NP cells derived from degenerative human disc. However, cell morphology was evidently altered, whereas apoptosis was not decreased following transfection. Further research is required to determine whether survivin may be used as a candidate for gene therapy and, more specifically, for the deceleration of the degeneration of NP cells, as well as to elucidate the mechanisms involved in this process.

## Figures and Tables

**Table I:** Demographic data of the patients.

Patient no.	Gender	Age (years)	Location of lumbar disc herniation	Pfirrmann grade
1	Male	46	L4/L5	IV
2	Female	50	L5/S1	V
3	Male	57	L4/L5	V
4	Male	61	L5/S1	V
5	Female	62	L4/L5	V
6	Female	55	L4/L5	IV
7	Male	48	L5/S1	IV
8	Male	53	L5/S1	V
9	Female	51	L4/L5	IV
10	Male	55	L4/L5	V

The intervertebral discs were classified acording to the Pfirrmann grade grading system for lumbar intervertebral disc degeneration.

**Table II:** Nucleotide sequences of sense and antisense primers and product size.

Gene	Primer	Product size (bp)
Survivin	F: CAGATGACGACCCCATAGAGGA	141
	R: CCTTTGCAATTTTGTTCTTGGC	
GAPDH	F: GGATTTGGTCGTATTGGG	205
	R: GGAAGATGGTGATGGGATT	

F, forward; R, reverse; GAPDH, glyceraldehyde-3-phosphate dehydrogenase.
